# A peptide derived from the amino terminus of leptin improves glucose metabolism and energy homeostasis in myotubes and db/db mice

**DOI:** 10.1016/j.jbc.2024.107919

**Published:** 2024-10-28

**Authors:** Mehmood Ali, Arvind Gupta, Rahul Dev Verma, Sariyah Akhtar, Jimut Kanti Ghosh

**Affiliations:** 1 Biochemistry and Structural Biology Division, CSIR-Central Drug Research Institute, Lucknow, India; 2 Academy of Scientific and Innovative Research (AcSIR), Ghaziabad, India

**Keywords:** leptin hormone, glucose metabolism, energy homeostasis, antidiabetic peptides, mitochondrial biogenesis

## Abstract

Leptin is an adipokine, which plays key roles in regulation of glucose metabolism and energy homeostasis. Therefore, identification of a short peptide from leptin which improves glucose-metabolism and energy-homeostasis could be of significant therapeutic importance. Mutational studies demonstrated that N-terminal of human leptin hormone is crucial for activation of leptin-receptor while its C-terminal seems to have lesser effects in it. Thus, for finding a metabolically active peptide and complimenting the mutational studies on leptin, we have identified a 17-mer (leptin-1) and a 16-mer (leptin-2) segment from its N-terminal and C-terminal, respectively. Consistent with the mutational studies, leptin-1 improved glucose-metabolism by increasing glucose-uptake, GLUT4 expression and its translocation to the plasma membrane in L6-myotubes, while leptin-2 was mostly inactive. Leptin-1-induced glucose-uptake is mediated through activation of AMPK, PI3K, and AKT proteins since inhibitors of these proteins inhibited the event. Leptin-1 activated leptin-receptor immediate downstream target protein, JAK2 reflecting its possible interaction with leptin-receptor while leptin-2 was less active. Furthermore, leptin-1 increased mitochondrial-biogenesis and ATP-production, and increased expression of PGC1α, NRF1, and Tfam proteins, that are important regulators of mitochondrial biogenesis. The results suggested that leptin-1 improved energy-homeostasis in L6-myotubes, whereas, leptin-2 showed much lesser effects. In diabetic, db/db mice, leptin-1 significantly decreased blood glucose level and improved glucose-tolerance. Leptin-1 also increased serum adiponectin and decreased serum TNF-α and IL-6 level signifying the improvement in insulin-sensitivity and decrease in insulin-resistance, respectively in db/db mice. Overall, the results show the identification of a short peptide from the N-terminal of human leptin hormone which significantly improves glucose-metabolism and energy-homeostasis.

Type 2 diabetes mellitus is a metabolic disease which is characterized by persistent increase in blood glucose level due to disturbance in glucose and lipid metabolism (1). Insulin resistance (IR) is one of the major pathophysiological causes of diabetes which occurs due to nonfunctioning of insulin signaling pathway, resulting in the gradual incidence of insulin deficiency (2, 3, 4). In addition to IR, inactivity of proteins and signaling pathways also lead to the inhibition of different physiological activities like GLUT4 translocation to plasma membrane (PM), glucose uptake into the cells, inhibition of glycogenesis, increase in gluconeogenesis, increase in fatty acid synthesis, and inhibition of β-oxidation of fatty acids (5, 6, 7). Abnormality in these physiological activities adds to hyperglycemia and diabetes (8). Further, diabetes is also associated with dysregulation in functioning of mitochondria and inhibition of mitochondrial biogenesis, leading to imbalance in energy expenditure and deficiency (9).

Leptin is an important adipokine hormone which improves glucose metabolism and energy homeostasis (10). It is a 16 kDa peptide hormone primarily expressed by white adipose tissue, and small amount of it is also expressed in placenta, ovary, skeletal muscle, pituitary gland, and lymphoid tissue (11). It is commonly known as obesity hormone because of its involvement in regulation of body weight and adipose tissue formation (12). Beside that leptin also regulates different other physiological activities *i.e.*, it reduces food intake, lowers appetite, improves glucose metabolism, increases insulin sensitivity, and improves energy homeostasis (10).

Leptin acts peripherally by binding to leptin receptors (LRs) in different peripheral tissues thus showing a direct and localized effect (13). It also acts systemically through the central nervous system *via* the LRs in the arcuate nucleus of the hypothalamus (14). Activation of leptin signaling in the hypothalamus and stimulation of neuroendocrine axis occurs through the longest form of LR-b, which is predominantly expressed in the brain. However in addition to LR-b, other spliced isoforms of LRs are largely expressed and are involved in activation of the leptin signaling in peripheral tissues (14, 15, 16).

LR transduces its downstream signal through activation of the JAK/STAT pathway and its metabolic effect is exerted *via* activation of the IRS1/PI3K/AKT and AMPK/ACC signaling pathways, which are involved in glucose and lipid metabolism (13, 17, 18). Activation of the PI3K/AKT and AMPK signaling improves blood glucose clearance by increasing glucose uptake and GLUT4 translocation (19). AMPK regulates lipid metabolism by activating β-oxidation of fatty acid and inhibiting fatty acid synthesis through inhibition of acetyl-CoA carboxylase (ACC) (20). Further, leptin improves insulin sensitivity and also increases expression and secretion of insulin (21, 22). AMPK acts as an energy sensor and effect of leptin on energy expenditure and homeostasis is exerted *via* activation of AMPK protein (23). Activation of AMPK increases energy expenditure, mitochondrial functioning, and its biogenesis (24) *via* activation of PGC1α, which is an important transcription factor that increases the expressions of Tfam and NRF1/2 genes. These two genes further increase the expressions of mitochondrial genes (24, 25). Due to its involvement in glucose metabolism and energy expenditure, deficiency of leptin or its resistance causes morbid obesity and diabetes (11). Considering the versatile physiological roles of leptin, investigations on the effects of leptin-derived peptides on physiological activities related to this hormone and the activation of associated signaling events are being carried out (26, 27). The optimism is that these studies could lead to the discovery of therapeutic peptides possessing the ability to improve glucose and lipid metabolism and energy homeostasis.

Leptin consists of 146 amino acids (28), and its crystal structure shows that it has four helical regions, helix-A, B, C, and D and two loop regions connecting the helix-A and B and helix-C and D (29). Synthetic peptides derived from different helical regions of leptin have been investigated for their activity by different groups (26, 27). Investigation on the biological activity of leptin-deletion analogs suggests that the N-terminal region [amino acid (a.a.) 22 to a.a. 115, 94 amino acid residues] of leptin is crucial for both receptor binding and its activation, while the C-terminal amino acid region (a.a. 116 to a.a. 166, 51 amino acid residues) does not possess any significant effect on the activation of LR in both *in vitro* and *in vivo* studies (30). These results suggest the possibility of finding a peptide from the N-terminal of leptin that could activate LR. Results indicate that amino acid region 39 to 42 in the AB loop is essential for LR activation (31). Point mutagenesis studies also suggested few critical amino acid residues (F41 and H46) in the loop region connecting the helix-A and B toward the N-terminal of leptin hormone (LH) (32) in receptor binding and activation. Different studies have already investigated the biological activities of the peptides derived from the helical regions of the LH (26, 27), while we did not find any study related to the peptides derived from the loop regions of leptin. Thus, to identify a peptide from leptin with metabolic properties, we selected a 17-mer segment (leptin-1) from its N-terminal loop region that encompasses the amino acid (a.a.) region 33 to 49. Further, we also intended to assess the activity of a similar sized peptide from the C-terminal region of LH which apparently possesses lesser role in leptin-receptor activation and related biological activities. For this purpose, we synthesized and characterized a 16-residue segment (leptin-2) corresponding to the amino acid region 123 to 138 of helix D.

We have primarily focused on exploring the effect of leptin-derived peptides in different signaling pathways involved in glucose metabolism, mitochondrial function and biogenesis, and oxidative phosphorylation in L6 myotubes. Additionally, we also investigated if these peptides can improve glucose metabolism in db/db mice. Remarkably, consistent with previous mutational studies (30), we observed significant metabolic properties of leptin’s N-terminal derived peptide, leptin-1 while the similar sized, C-terminal derived peptide, leptin-2 was much less active.

## Results

### Identification of leptin-1 and leptin-2 peptides from the adipokine hormone leptin

**Figure 1:**
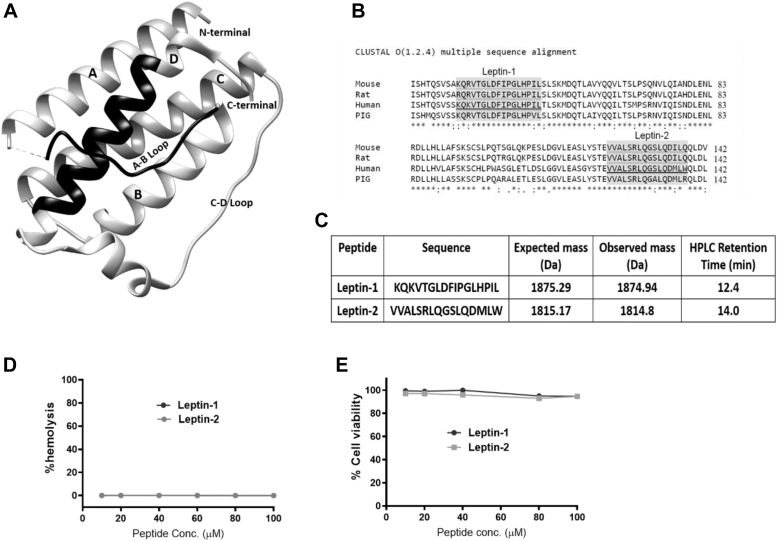
**Identification of peptide sequences from human leptin.***A*, 3D structures of human leptin hormone obtained from PDB (44). The selected peptide sequences are highlighted, leptin-1 in A-B loop region toward the N-terminal and leptin-2 in helix-D of LH toward the C-terminal are highlighted in *black color*. *B*, multiple sequence alignments of leptin-1 and leptin-2 were done using the online tool CLUSTAL omega. *C*, synthesized peptide sequences along with their expected and observed molecular masses and HPLC retention times. *D*, hemolytic activity of the peptides against human RBCs (hRBCs). *E*, cytotoxicity study of leptin-1 and leptin-2 against mammalian cell line L6 as presented with respect to the viability of these cells with respect to the concentrations of each peptide. LH, leptin hormone; PDB, Protein Data Bank.

Leptin is an important peptide hormone which plays crucial role in energy homeostasis, glucose metabolism, food intake, innate immunity, and fertility. Metabolic effect of leptin is exerted through activation of AMPK and PI3K/AKT signaling pathways (13). Binding of leptin to LR activates the JAK/STAT signaling pathway, which in-turn activates the downstream AMPK and PI3K/AKT proteins. Crystal structure of LH consists of four helical regions *i.e.*, helix-A, B, C, and D and two loop regions one connecting the helix-A and B and another connecting the helix-C and D (Fig. 1*A*). The binding affinity of leptin to LR and its ability to activate the receptor varies among different regions of leptin. Based on the ability of different regions of leptin in receptor binding and activation, considering the importance of conserved domain (Fig. 1*B*) and the critical amino acid residues of LH in its activity as mentioned in the “Introduction section”, we have selected a 17-residue protein segment (amino acid 33–49) from the N-terminal loop region connecting helix-A and B and a 16-residue segment (amino acid 123–138) from the C-terminal helix-D of LH. It is worthy to mention that while N-terminal, leptin-1 possesses two or more lysine, leucine, isoleucine, and proline residues and single valine, aspartic acid, phenylalanine, and histidine residues, C-terminal, leptin-2 comprises two or more valine and leucine residues and single alanine, arginine, methionine, and tryptophan residues that often occur in protein-protein and protein-peptide interfaces (33) which could be conducive for the interaction of the corresponding peptide with its target protein. The protein segments, a.a. 33 to 49 (leptin-1) and a.a. 123 to 138 (leptin-2) were then synthesized by solid phase peptide synthesis method and purified using reverse phase-high performance liquid chromatography. Identity of the synthesized peptides was confirmed by determining their molecular masses through MALDI-MS analyses (Figs. 1*C* and S1). The purified peptides were then investigated for their toxicity and biological properties related to LH in L6-myotubes and diabetic animal models.

### Leptin-1 and leptin-2 peptides are nonhemolytic to hRBCs and nontoxic to L6 cells

*In vitro* toxicity of leptin-1 and leptin-2 peptides was assessed by studying the hemolytic and cytotoxic effects of the peptides on hRBCs and mammalian cell line L6, respectively. Both the peptides did not show any hemolysis of hRBCs (Fig. 1*D*) and cytotoxicity to L6 cells (Fig. 1*E*) up to a concentration of 100 μM. L6 cells showed ∼95% viability in presence of leptin-1 and leptin-2 (Fig. 1*E*).

### Leptin-1 increased glucose uptake in time and dose-dependent manner in L6 myotubes

**Figure 2:**
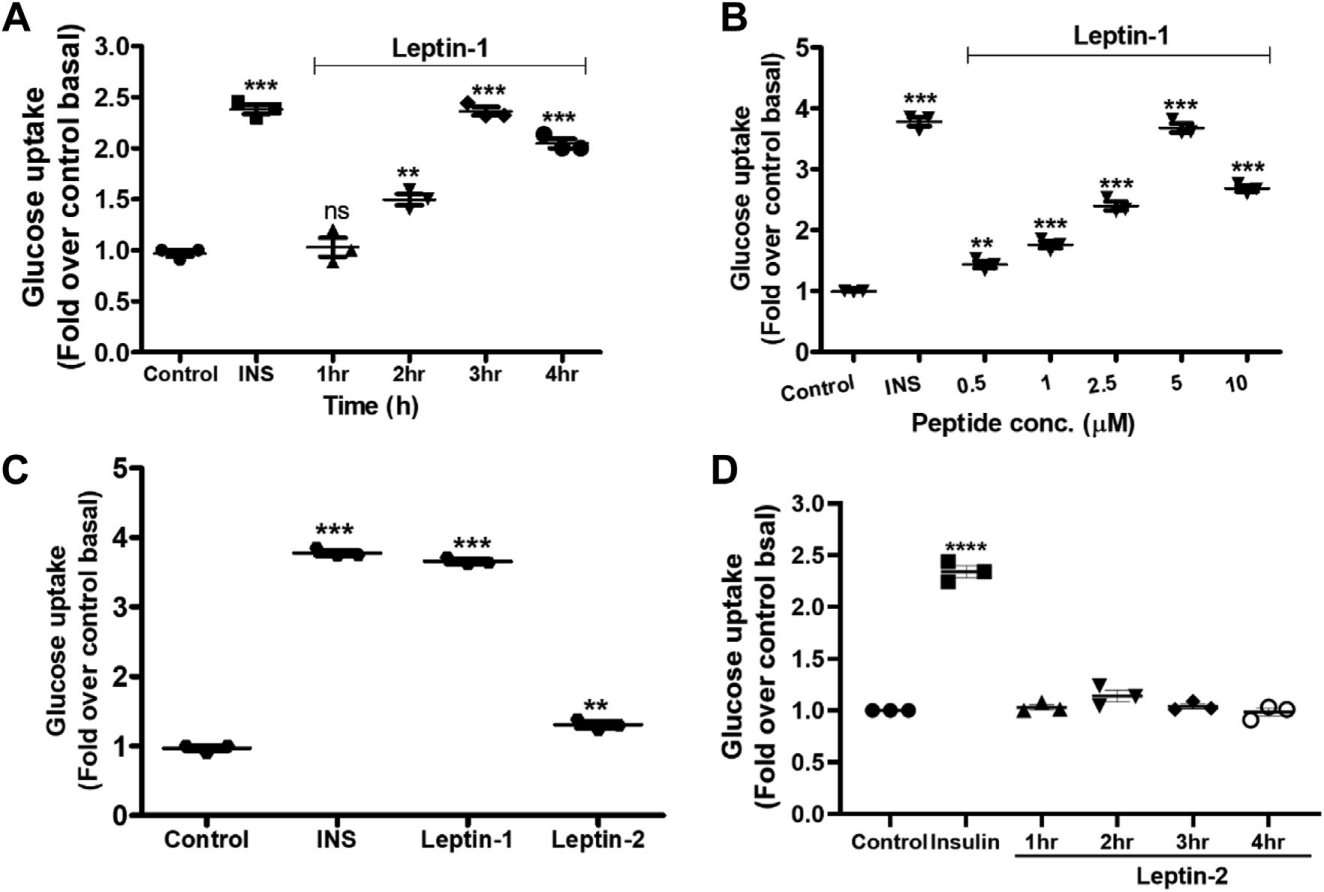
**Leptin derived peptide Leptin-1 but not Leptin-2 induced glucose uptake in L6 myotubes.***A*, time-dependent increase in glucose uptake in L6 myotubes induced by leptin-1 peptide, no significant increase was observed at 1 h incubation, while 2 h incubation showed 1.5-fold increase in glucose uptake. Maximum increase was observed at an incubation time of 3 h. *B*, dose-dependent increase of glucose uptake in L6 myotubes induced by leptin-1 peptide, maximum increase in glucose uptake was observed at leptin-1 concentration of 5 μM. (∗∗*p* ≤ 0.001; ∗∗∗*p* ≤ 0.001). *C*, comparative glucose uptake induced by leptin-1 and leptin-2 peptides in L6-myotubes. Compared to the leptin-2 (5 μM), leptin-1 (5 μM) peptide showed more significant increase in glucose uptake in L6 myotubes. (∗∗*p* ≤ 0.001; ∗∗∗*p* ≤ 0.0001). *D*, time-dependent glucose uptake analysis in the presence of leptin-2 peptide (20 μM) at 1, 2, 3, and 4 h. Data show that leptin-2 could not induce glucose uptake in L6-myotubes at a concentration of 20 μM when incubated for different time periods. Positive control insulin (100 nM) significantly increased glucose uptake (∗∗∗∗*p* ≤ 0.0001).

To investigate the metabolic effect of leptin-derived peptides, leptin-1 and leptin-2, glucose uptake assays were performed in L6 myotubes by incubating the cells with these two peptides. For time-dependent effect of the peptides on glucose uptake, differentiated L6 myotubes were incubated with leptin-1 (20 μM) for different time periods of 1, 2, 3, and 4 h. After incubation, glucose uptake was measured in the peptide-treated myotubes using 2NBDG (200 μM) glucose analogue. Interestingly, leptin-1 peptide significantly increased glucose uptake in L6 myotubes (Fig. 2*A*). A significant increase in glucose uptake in presence of leptin-1 was observed at 2 h incubation time, however; maximum increase in glucose uptake was detected at an incubation period of 3 h (Fig. 2*A*). After investigating the time-dependent effect, dose dependent effect of leptin-1 peptide on glucose uptake was investigated in the same cells. For this, L6 myotubes were incubated with leptin-1 peptide at different concentrations of 0.5, 1, 2.5, 5, and 10 μM for 3 h and glucose uptake assays were performed. We observed that leptin-1 significantly increased glucose uptake in L6 myotubes in a dose-dependent manner. A significant increase was observed at 0.5 μM peptide concentration, and it increased concomitantly with increase in peptide dose (Fig. 2*B*). A maximum increase of 3.6-fold in glucose uptake was observed at 5 μM concentration, beyond which glucose uptake became saturated and did not increase further (Fig. 2*B*). These data suggested that the N-terminal derived peptide, leptin-1 significantly increased glucose uptake in L6 myotubes. Further, maximum increase in glucose uptake was observed at an incubation period of 3 h and at a peptide concentration of 5 μM. Following this, all further experiments were carried out with the peptide concentration of 5 μM and an incubation period of 3 h.

### Leptin-2 peptide did not show any significant increase in glucose uptake in L6 myotubes

To investigate the effect of leptin-2 on glucose uptake, differentiated myotubes were incubated with leptin-1 and leptin-2 peptides at similar concentration of 5 μM and incubation period of 3 h, and performed glucose uptake assay. Data suggested that leptin-2 treatment showed the least increase in glucose uptake of 1.2-folds (Fig. 2*C*). Whereas, leptin-1 treated L6-myotubes showed significant increase in glucose uptake by 3.6-folds (Fig. 2*C*). Further, we have also investigated if the 16-mer peptide, leptin-2, could increase glucose uptake at higher peptide concentration and different time periods. For examining this possibility, we incubated L6-myotubes with leptin-2 peptide at a high concentration of 20 μM for different time periods of 1, 2, 3, and 4 h and performed glucose uptake assays. Data suggested that leptin-2 did not show any significant increase in glucose uptake even at a peptide concentration of 20 μM and at different incubation period till 4 h (Fig. 2*D*). Hence, from the comparative peptide-induced glucose uptake assay in presence of leptin-1 and leptin-2 peptides at different time intervals, it appears that leptin-2 does not show appreciable glucose uptake in L6 myotubes while as shown before, leptin-1 is significantly active in this regard.

### Leptin-1 enhanced GLUT4 translocation to the PM of L6 myotubes

**Figure 3:**
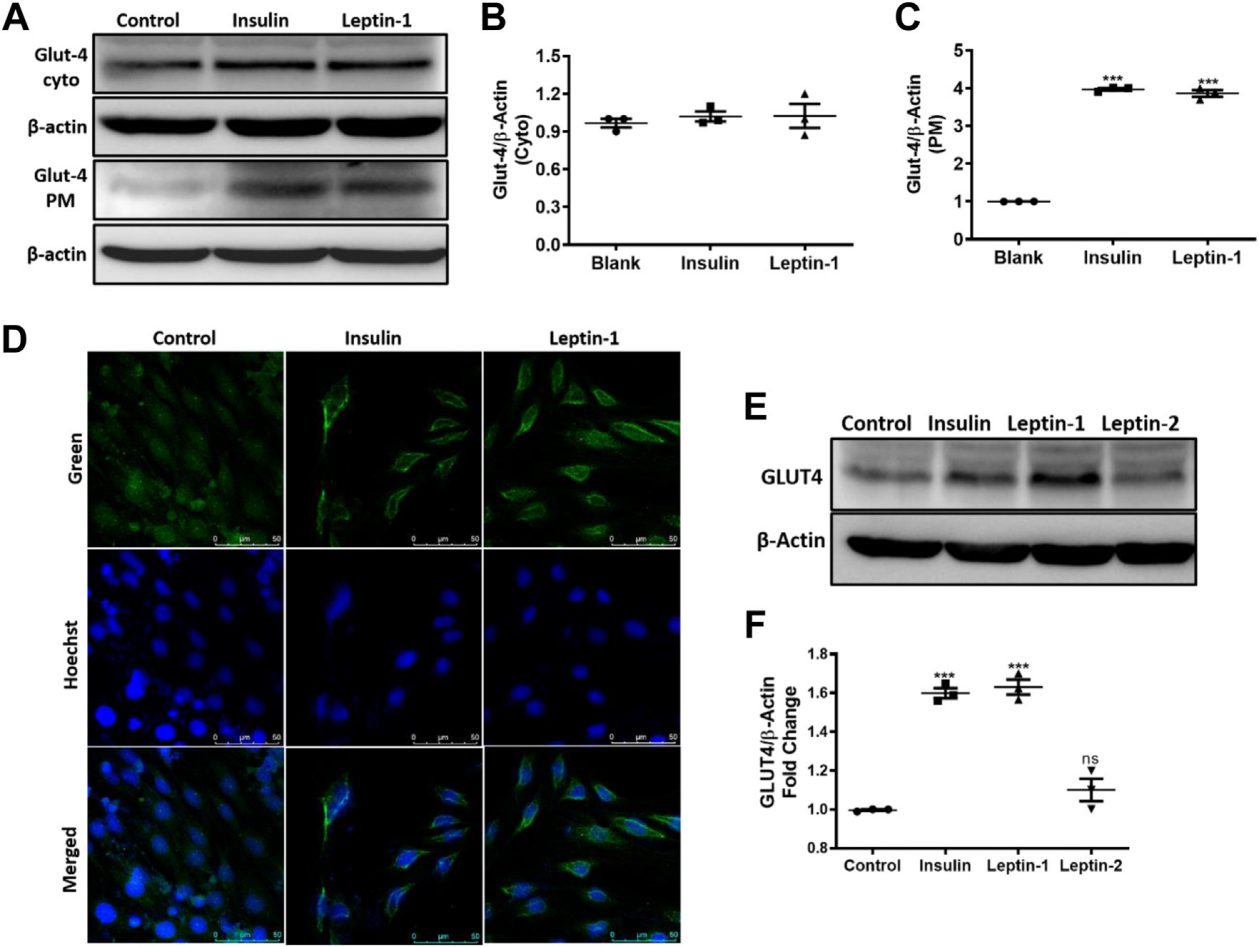
**Leptin-1 induced GLUT4 translocation to PM and increased its expression.***A*, Western blot images showing GLUT4 translocation to PM in L6 myotubes, cytosolic, and membrane fraction of GLUT4 protein in myotubes treated with vehicle, insulin(100 nM) and leptin-1 peptide (5 μM). Leptin-1 increased GLUT4 translocation to PM by 3.8-fold and insulin increased by 4-fold compared to the vehicle treated negative control. *B* and *C*, quantification of the Western blot bands, cytosolic and membrane fractions of GLUT4. *D*, immunocytochemistry, confocal imaging of cells showing GLUT4 translocation in L6 myotubes treated with vehicle, insulin (100 nM) and leptin-1 peptide (5 μM). Leptin-1 and insulin significantly increased GLUT4 translocation to PM compared to vehicle-treated negative control. Increased green fluorescence on the membranes of the L6 myotubes in leptin-1 and insulin-treated samples represent the translocated GLUT4 proteins. *E*, Western blot images showing expression of GLUT4 protein in L6 myotubes in the presence of leptin-1, leptin-2, and insulin. *F*, quantification of the GLUT4 Western blot bands. (∗∗∗*p* ≤ 0.0001). Expressions of total proteins in A and E were quantified with loading control beta-actin. PM, plasma membrane.

GLUT4 is the final effector protein in insulin-induced glucose uptake; however, it remains bound to lipid vesicles in the cytosol under basal condition. Upon stimulation, it translocates to the PM and transport glucose into the cells. Therefore, we examined the effect of leptin-1 on GLUT4 translocation to PM in L6 myotubes. Differentiated myotubes were incubated with leptin-1 (5 μM) for 3 h and insulin (100 nM) for 20 min. Their cytosolic and membrane fractions were isolated separately and resolved on 10% SDS-PAGE and performed Western blot to estimate GLUT4 translocation to PM. Results showed that PM content of GLUT4 protein increased in leptin-1-treated myotubes as compared to the vehicle-treated negative control, suggesting an increased translocation of GLUT4 to PM (3.8-fold) (Fig. 3, *A–C*) in the presence of the peptide. Insulin, used as positive control also increased GLUT4 translocation by 4-fold (Fig. 3, *A–C*). These data suggested that leptin-1 significantly increased GLUT4 translocation to PM compared to the vehicle-treated negative control.

To further confirm the effect of leptin-1 on GLUT4 translocation, GLUT4 translocation was also assessed by immunocytochemistry (ICC) method in L6 myotubes. Differentiated L6-myotubes in 4-chambered slides were incubated separately with leptin-1 (5 μM) for 3 h and insulin (100 nM) and 20 min. Assay was performed according to the protocol described in experimental procedures. Similar to the findings observed in Western blot assay, ICC assay confirmed that leptin-1 significantly induced GLUT4 translocation to PM (Fig. 3*D*). Myotubes treated with leptin-1 peptide and insulin showed appreciable increase in green fluorescence on PM of the cells, which signifies an increase in GLUT4 translocation (Fig. 3*D*). Thus, from the Western blot and ICC assay data, we can conclude that leptin-1 peptide possesses the activity to increase GLUT4 translocation to the PM of L6-myotubes.

### Leptin-1 showed higher increase in GLUT4 expression as compared to leptin-2

After investigating the effect of leptin-1 on GLUT4 translocation, we next determined the effect of leptin-2 peptide on GLUT4 expression along with leptin-1 in L6 myotubes. L6 myotubes were treated with leptin-1 and leptin-2 at 5 μM concentration for 3 h. SDS-PAGE and Western blotting was performed on lysate prepared from the peptide treated cells. We observed that leptin-2 peptide did not show a significant increase in GLUT4 expression in L6 myotubes in comparison to leptin-1 (Fig. 3, *E* and *F*) which corroborated with the glucose uptake induced by both these peptides as shown in previous sections.

### Leptin-1 exerts its metabolic effect through the activation of Janus Kinase-2 (JAK2) protein

**Figure 4:**
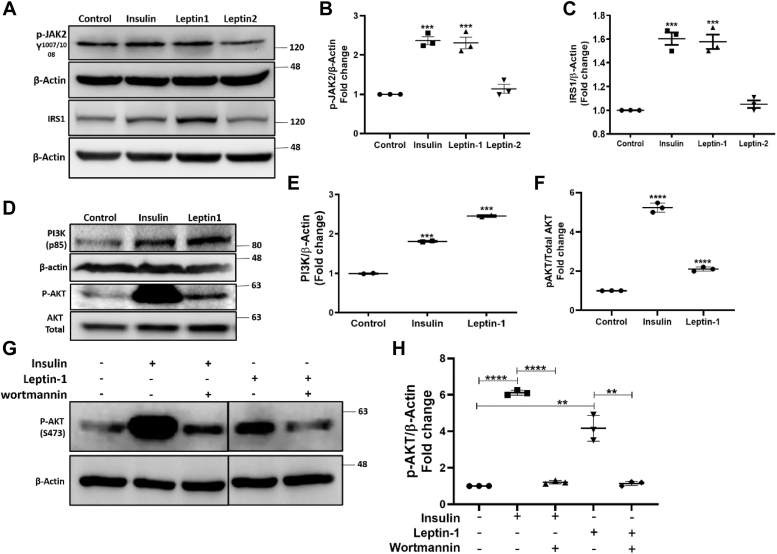
**Leptin-1 activates JAK2 and its glucose lowering effect is mediated through activation of IRS1/PI3K/AKT signaling axis.***A*, activation of JAK2 protein in the presence of leptin-1 and leptin-2 peptides. Western blot shows the phosphorylation level of its Tyr^1007/1008^residue. Leptin-1 induced phosphorylation of JAK2 whereas Leptin-2 showed much less activity. A also presents Western blot images showing expression of IRS1 in L6 myotubes in presence of leptin-1 and leptin-2 peptides and insulin. *B* and *C*, respectively show quantification of JAK2 and IRS1 Western blot bands with respect to beta-actin loading control. *D*, Western Blots showing expressions of PI3K and p-AKT proteins in leptin-1 treated L6 myotubes. *E*, quantification of the PI3K with respect to beta-actin loading control. *F*, quantification of p-AKT with respect to total AKT. *G*, Western blot images showing activation of p-AKT in the presence of insulin and leptin-1 peptide and parallelly showing its inhibition in L6 myotubes pretreated with wortmannin. The *vertical line* in the blot indicates the cut and rejoining the same blot after excluding a band for presentation purpose. *H*, quantification plot for p-AKT bands in the presence of PI3K inhibitor, wortmannin in the Western blot images with respect to beta-actin. All the Western blot experiments were repeated thrice, only data with *p*-value ≤ 0.05 were considered significant. (∗*p*-value ≤ 0.01; ∗∗*p*-value ≤ 0.001; ∗∗∗*p*-value ≤ 0.0001). JAK2, Janus Kinase-2.

LH exerts its metabolic effect through the activation of JAK2 protein, which is an important downstream target of LR. Thus, to elucidate, whether leptin-derived peptides follow a pathway similar to its parent protein leptin, we investigated the effect of leptin-1 and leptin-2 on the activation of JAK2 protein. For this, L6-myotubes were incubated with leptin-1 and leptin-2 peptides at 5 μM concentration and an incubation time of 3 h as used for other experiments. Whole cell lysate was prepared and the activation of JAK2 was examined by Western blotting. Remarkably, we observed that leptin-1 peptide significantly activated JAK2 protein in L6-myotubes (Fig. 4, *A* and *B*). JAK2 Activation was accessed by quantifying the phosphorylation of the tyrosine residue at 1007/1008 (Tyr^1007/1008^) position. Phosphorylation level of Tyr^1007/1008^ increases with the activation of the LR. Thus, interestingly the peptide derived from the N-terminal of LH, leptin-1 increased the phosphorylation level of Tyr^1007/1008^ residue by ≈1.5-folds whereas, its C-terminal derived peptide, leptin-2, only increased the Tyr^1007/1008^ phosphorylation by ≈1.1-folds (Fig. 4, *A* and *B*). Insulin, used as a positive control, increased Tyr^1007/1008^ phosphorylation by ≈1.5-folds (Fig. 4, *A* and *B*). Thus, from these data we could infer that leptin-1 derived from the N-terminal of LH significantly activated the leptin-receptor substrate JAK2. However, the C-terminal derived, leptin-2 showed relatively much lesser effect than leptin-1.

### Leptin-1 activated the IRS1/PI3K/AKT/GLUT4 signaling pathway in L6 myotubes

LH is known to activate the IRS1/PI3K/AKT signaling pathway which is involved in glucose metabolism (18, 20). Therefore, to examine the involvement of IRS1/PI3K/AKT proteins in leptin-1-induced signaling pathways toward glucose uptake, we determined their activation by looking into their expressions in L6 myotubes in presence of leptin-1 by Western blotting. For this purpose, differentiated L6 myotubes were treated with 5 μM of leptin-1 peptide for 3 h and positive control, insulin at 100 nM for 30 min and negative control was treated with vehicle only. Western blot analysis of the whole cell lysate prepared from the leptin-1 treated cells suggested that leptin-1 peptide significantly increased the activation and expression of IRS1 (1.61-folds), PI3K (2.4-folds) and AKT (2.8-folds) (Fig. 4, *A*, *C–F*) and GLUT4 (1.61-folds) (Fig. 3, *E* and *F*) proteins in L6 myotubes as compared to the vehicle-treated negative control. Insulin also increased activation of IRS1, PI3K, AKT, and GLUT4 protein by 1.6-folds, 1.8-folds, 4.2-folds, and 1.6 folds, respectively (Fig. 4, *A–F*). In parallel, the effect of leptin-2 peptide on activation of IRS1 and GLUT4 was also examined in L6 myotubes. We observed that leptin-2 peptide showed lesser increase in the expression of IRS1 and GLUT4 proteins than leptin-1 and insulin-treated myotubes (Figs. 3*E* and 4, *A* and *C*). The results suggest that leptin-1 activates IRS1, PI3K, AKT, and GLUT4 in L6 myotubes.

### Leptin-1-induced activation of p-AKT was inhibited in presence of PI3K inhibitor wortmannin

We observed that leptin-1 increased the activation of PI3K and AKT proteins. Both PI3K and AKT are important proteins involved in insulin-induced glucose uptake and AKT activation is increased in presence of the activated PI3K protein (19). Therefore, we investigated whether the activation of AKT by leptin-1 is mediated through PI3K. For this, we determined the activation of p-AKT by leptin-1 peptide in L6 myotubes in the presence of PI3K-inhibitor, wortmannin. L6 myotubes were pretreated with wortmannin (200 nM) for 1 h before leptin-1 peptide treatment for 3 h. Whole cell lysate was prepared and p-AKT activation in all samples were examined by Western blotting. Data showed that insulin and leptin-1 peptide treatment significantly increased p-AKT activation (Fig. 4, *G* and *H*). However, p-AKT activation by leptin-1 peptide was inhibited in wortmannin pretreated L6 myotubes (Fig. 4, *G* and *H*). Thus, the results signify that leptin-1-induced activation of p-AKT is mediated through PI3K protein and its activation is diminished in presence of PI3K inhibitor.

### Leptin-1 activates AMPK protein by increasing its Thr-172 phosphorylation in L6 myotubes

**Figure 5:**
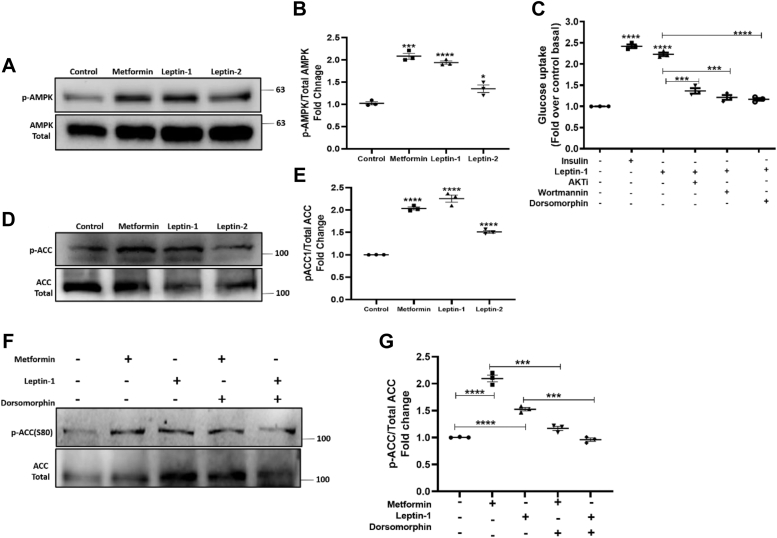
**Leptin-1 also activates AMPK protein.***A*, activation of p-AMPK (Thr172 phosphorylation) in L6 myotubes treated with leptin-1, leptin-2, and metformin as positive control. *B*, quantification of the p-AMPK bands by normalizing with total AMPK. *C*, effect of PI3K, AKT, and AMPK inhibitors on leptin-1 induced glucose uptake in L6 myotubes. Preincubation of L6 myotubes with AMPK, PI3K, and AKT inhibitors prior to leptin-1 treatment inhibited leptin-1 induced glucose uptake in L6 myotubes treated with wortmannin, AKTi, and dorsomorphin. *D*, phosphorylation of acetyl-CoA carboxylase-2 (ACC2) at its S80 residue, an indicator of its inactivation, in the presence of leptin-1 and leptin-2 peptides and metformin, used as positive control. (∗∗∗*p* ≤ 0.0001). *E*, quantification of the p-ACC Western blot bands with respect to total ACC. *F*, Western blot images showing leptin-1-induced phosphorylation of ACC2, is reduced in presence of AMPK inhibitor, dorsomorphin, suggesting p-ACC2 inhibition by leptin-1 is mediated through AMPK. *G*, quantification of the p-ACC2 Western blot band. Quantification of the phosphoproteins was done with their total protein.

LH-associated glucose metabolism involves AMPK activation. Therefore, we investigated whether leptin-1 and leptin-2 increase the activation of AMPK protein. For this purpose, L6 myotubes were treated with 5 μM concentration of leptin-1 and leptin-2 peptides for 3 h and positive control, metformin (50 μM) for 6 h. Activation of AMPK protein was assessed by determining the phosphorylation level of its Thr-172 residue by Western blot. We observed that leptin-1 significantly activated AMPK protein in L6 myotubes and increased phosphorylation of its Thr-172 residue by 1.9-fold (Fig. 5, *A* and *B*), whereas leptin-2 showed much lesser effect in AMPK activation (Fig. 5, *A* and *B*). Metformin increased AMPK Thr-172 phosphorylation by 2-folds (Fig. 5, *A* and *B*). Hence these data suggested that leptin-1 significantly increased AMPK activation compared to the only vehicle-treated negative control and leptin-2 peptide in L6 myotubes, which further confirmed that the glucose uptake induced by leptin-1 peptide was associated with activation of AMPK protein in L6 myotubes.

### Leptin-1-induced glucose uptake is mediated through activation of AMPK and PI3K/AKT signaling axis

Leptin-1 augmented the expressions of PI3K, AKT, and AMPK in L6 myotubes as described in the previous section. Therefore, to investigate the involvement of these proteins in leptin-1-induced glucose uptake, we determined the effect of inhibitors of PI3K, AKT, and AMPK proteins on leptin-1-induced glucose uptake in L6 myotubes. The differentiated myotubes were pretreated with PI3K-inhibitor, wortmannin (200 nM), AKT-inhibitor, AKTi (420 nM) and AMPK-inhibitor, dorsomorphin (109 nM) for 1 h prior to peptide treatment. The cells were then treated with 5 μM of leptin-1 peptide for 3 h and 100 nM of insulin for 30 min as a positive control. Glucose uptake assay in these inhibitor and peptide treated cells suggested that leptin-1 induced glucose-uptake in L6 myotubes were inhibited in presence of wortmannin, AKTi, and dorsomorphin (Fig. 5*C*). As expected, only leptin-1 and insulin treated myotubes showed increased glucose uptakes of 2.2-fold and 2.4-fold, respectively (Fig. 5*C*). However, pretreatment of L6 myotubes with PI3K, AKT, and AMPK inhibitors reduced leptin-1-induced glucose uptake to ∼1.2-folds (Fig. 5*C*). Thus, the results suggest that leptin-1-induced glucose uptake in L6 myotubes is mediated through activation of PI3K, AKT, and AMPK proteins.

### Leptin-1 inhibited acetyl-CoA carboxylase-2 by phosphorylating its serine-80 residue

ACC2 is a downstream target of AMPK protein, and is inhibited by AMPK thereby inhibiting fatty acid synthesis. Upon activation of AMPK protein, it causes inhibition of ACC2 by phosphorylating at its serine-80 (S80) residue. Since leptin-1 significantly increased the activation of AMPK (Fig. 5, *A* and *B*), we also investigated whether leptin-1 was able to inhibit ACC2 by determining the phosphorylation level of its S80 residue in the presence of leptin-1. Cells were incubated with leptin-1 (5 μM) and leptin-2 (5 μM) for 3 h, and lysate was prepared for downstream expression analysis by Western blotting. Western blot data suggested that leptin-1 significantly increased phosphorylation of the S80 residue of ACC2 protein (Fig. 5, *D* and *E*) signifying its inhibition in presence of leptin-1. However, leptin-2 did not increase the phosphorylation of S80 residue of ACC2 (Fig. 5, *D* and *E*). Positive control, metformin also significantly increased S80 residue phosphorylation (Fig. 5, *D* and *E*). Thus, these data suggested that N-terminal leptin derived peptide, leptin-1 possesses the ability to activate AMPK protein and thereby increases phosphorylation of the S80 residue of ACC2, indicating its inhibition. Whereas, leptin-2 peptide showed relatively less effect on inhibition of ACC2.

### Inhibition of ACC2 by leptin-1 peptide is mediated through activation of AMPK

To further investigate if phosphorylation of the S80 residue of ACC in the presence of leptin-1 peptide is mediated through AMPK, we determined the effect of dorsomorphin (AMPK inhibitor) on leptin-1-induced phosphorylation at S80 residue of ACC2 in L6 myotubes. For this purpose, L6 myotubes were pretreated with dorsomorphin (109 nM) for 1 h and then incubated with leptin-1 (5 μM) for 3 h. Western blot analysis of the whole cell lysate prepared from these dorsomorphin and leptin-1 treated cells suggested that pretreatment with dorsomorphin prevented leptin-1 and metformin induced phosphorylation of S80 of ACC2 thereby preventing its inhibition in L6 myotubes (Fig. 5, *F* and *G*). Both leptin-1 and metformin alone treated cells showed a significant increase in S80 residue phosphorylation of ACC2 thereby inhibiting it (Fig. 5, *F* and *G*). Hence, these data suggested that phosphorylation of ACC2 at its S80 residue by leptin-1 peptide is mediated through activation of the AMPK protein.

### Leptin-1 increases mitochondrial biogenesis in L6 myotubes

**Figure 6:**
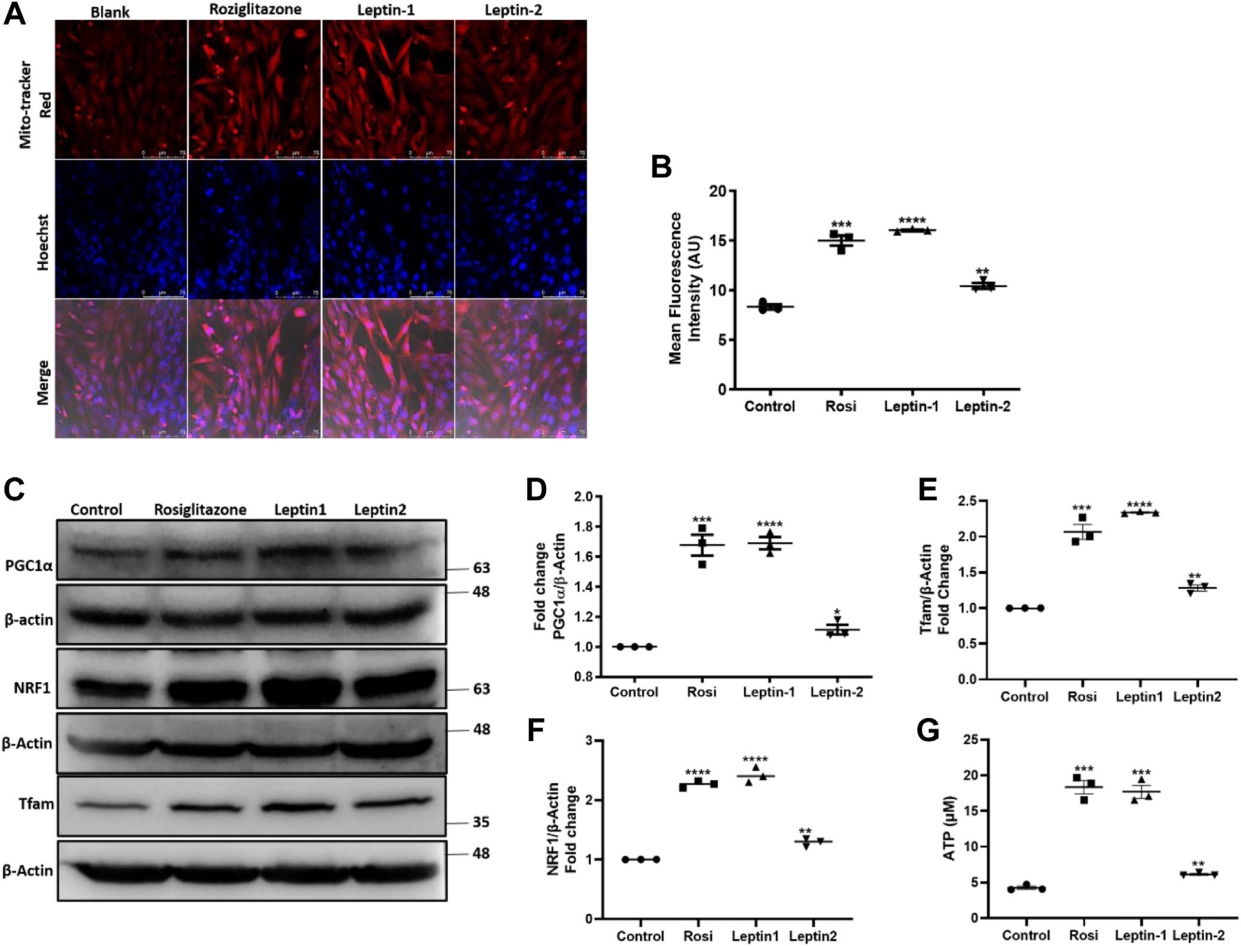
**Leptin-1 increased mitochondrial biogenesis and ATP production in L6 myotubes.***A*, confocal microscopy images showing increased red fluorescence in leptin-1 and rosiglitazone-treated cells, representing the increased mitochondrial biogenesis in L6 myotubes. *B*, mean fluorescence intensity from each sample was measured using ImageJ by selecting equal area from each image. *C*, Western blot images showing expression of PGC1α, NRF1, and Tfam in L6 myotubes in presence of rosiglitazone, leptin-1 and leptin-2. Significance of data was calculated using Graphpad prism software. *D*–*F*, histogram showing the quantification of the Western blot bands of PGC1α, NRF1, and Tfam. *G*, ATP production in L6 myotubes in presence of leptin-1 and leptin-2 peptides and rosiglitazone as assayed by ATP determination kit (Thermo Fisher Scientific). (∗*p*-value ≤ 0.05; ∗∗*p*-value ≤ 0.005; ∗∗∗*p*-value ≤ 0.0005; ∗∗∗*p*-value ≤ 0.0001). Expression of total proteins was quantified with loading control beta-actin.

One of the important physiological functions that often get disturbed in diabetes is the functioning and content of mitochondria which is directly involved in energy homeostasis. Different signaling pathways and proteins are involved in mitochondrial function and its biogenesis. AMPK is one among them, which is activated in energy deficient conditions and in turn increases the production of ATP. It activates different other proteins involved in mitochondrial functioning and biogenesis. Therefore, we further investigated the ability of leptin-1 peptide to induce mitochondrial biogenesis in L6 myotubes. L6 myotubes were incubated with leptin-1, leptin-2, and rosiglitazone (10 μM) for 24 h. Myotubes were then stained with Mitotracker red for 30 min, Hoechst nuclear staining for 5 min and the slides were fixed and imaged using confocal microscope. Confocal microscopy images showed that leptin-1 and rosiglitazone-treated myotubes took increased Mitotracker red stain as compared to the negative control sample suggesting that leptin-1 and rosiglitazone significantly increased mitochondrial biogenesis (Fig. 6*A*). The red Mitotracker stain is specific to mitochondria and the cells with high mitochondrial number take maximum stain. Similar to the inability of leptin-2 to induce glucose uptake and AMPK activation, the leptin-2 treated myotubes also stained less with red Mitotracker stain as compared to leptin-1 treated myotubes (Fig. 6*A*). The results suggested that, leptin-2 also had lesser effect on mitochondrial biogenesis in comparison to leptin-1 and rosiglitazone (Fig. 6*A*). The fluorescence intensity measurement using ImageJ software (https://imagej.net/ij/docs/install/windows.html#install) showed increased mitochondrial red fluorescence intensity in samples treated with leptin-1 and rosiglitazone while the leptin-2 treated myotubes showed less fluorescence intensity as compared to that of leptin-1. Negative control sample showed the least fluorescence intensity and the red fluorescence was directly proportional to mitochondrial biogenesis (Fig. 6*B*). Hence these results suggested that leptin-1 peptide significantly increased mitochondrial biogenesis in L6 myotubes.

### Leptin-1 induced expression of PGC1α, NRF1, and Tfam proteins that are important regulators of mitochondrial biogenesis

PGC1α is an important regulator of mitochondrial biogenesis which activates and increases the expression of different proteins like NRF1 and Tfam that are involved in functioning and biogenesis of mitochondria. After observing leptin-1-induced increase in mitochondrial biogenesis in L6 myotubes, we further investigated whether leptin-1 could also activate the proteins that are involved in the biogenesis of mitochondria. For this, L6 myotubes were incubated with leptin-1 and leptin-2 peptides for 3 h and whole cell lysates were prepared for analysis of expressions of PGC1α, NRF1, and Tfam proteins by Western blotting. Images showed that leptin-1 significantly increased the expression of PGC1α (1.7-folds), NRF1 (2.3-folds), and Tfam (2.4-folds) in L6 myotubes (Fig. 6, *C–F*). We also observed that the expressions of PGC1α, NRF1, and Tfam were more augmented when L6 myotubes were treated with leptin-1 as compared to that when the myotubes were treated with leptin-2 (Fig. 6, *C–F*). Data suggested that along with activating biogenesis of mitochondria, leptin-1 also stimulated the proteins (PGC1α, NRF1, and Tfam), involved in mitochondrial biogenesis. Further, the outcome of these assays also suggested higher metabolic properties of leptin-1 than leptin-2.

### Leptin-1 increased ATP production in L6 myotubes

Mitochondria are the powerhouse of the cells because of their ability to produce ATP. Previous results established the increase in mitochondrial biogenesis in the presence of leptin-1 peptide in L6-myotubes. Therefore, considering the potential of leptin-1 peptide to increase mitochondrial biogenesis and capability of mitochondria to produce ATP, we investigated whether mitochondrial biogenesis induced by leptin-1 was also associated with increased production of ATP in L6-myotubes. For this, L6-myotubes were incubated with leptin-1 and leptin-2 peptides and rosiglitazone (10 μM) as positive control for 24 h. ATP was extracted by following the procedures mentioned in the experimental section and its concentration was measured using the ATP detection kit. Interestingly, in concordance with the increase in mitochondrial biogenesis, leptin-1 treated L6-myotubes also showed an increased production of ATP (20 μM) (Fig. 6*G*), compared to the vehicle-treated negative control (4 μM). Previous results established that leptin-2 did not show any significant effect on glucose metabolism as compared to leptin-1 peptide. Consistent with that observation, leptin-2 peptide did not show any significant increase in ATP production (6 μM) as compared to leptin-1. Rosiglitazone, used as positive control, significantly increased ATP production (13 μM) (Fig. 6*G*). Hence, from these assays we could infer that the N-terminal LH-derived leptin-1 possesses significant activity to increase ATP production whereas, C-terminal derived, leptin-2 showed significantly lesser activity in ATP production in L6-myotubes.

### Leptin-1 did not show any toxic effect in Balb/c mice

**Figure 7:**
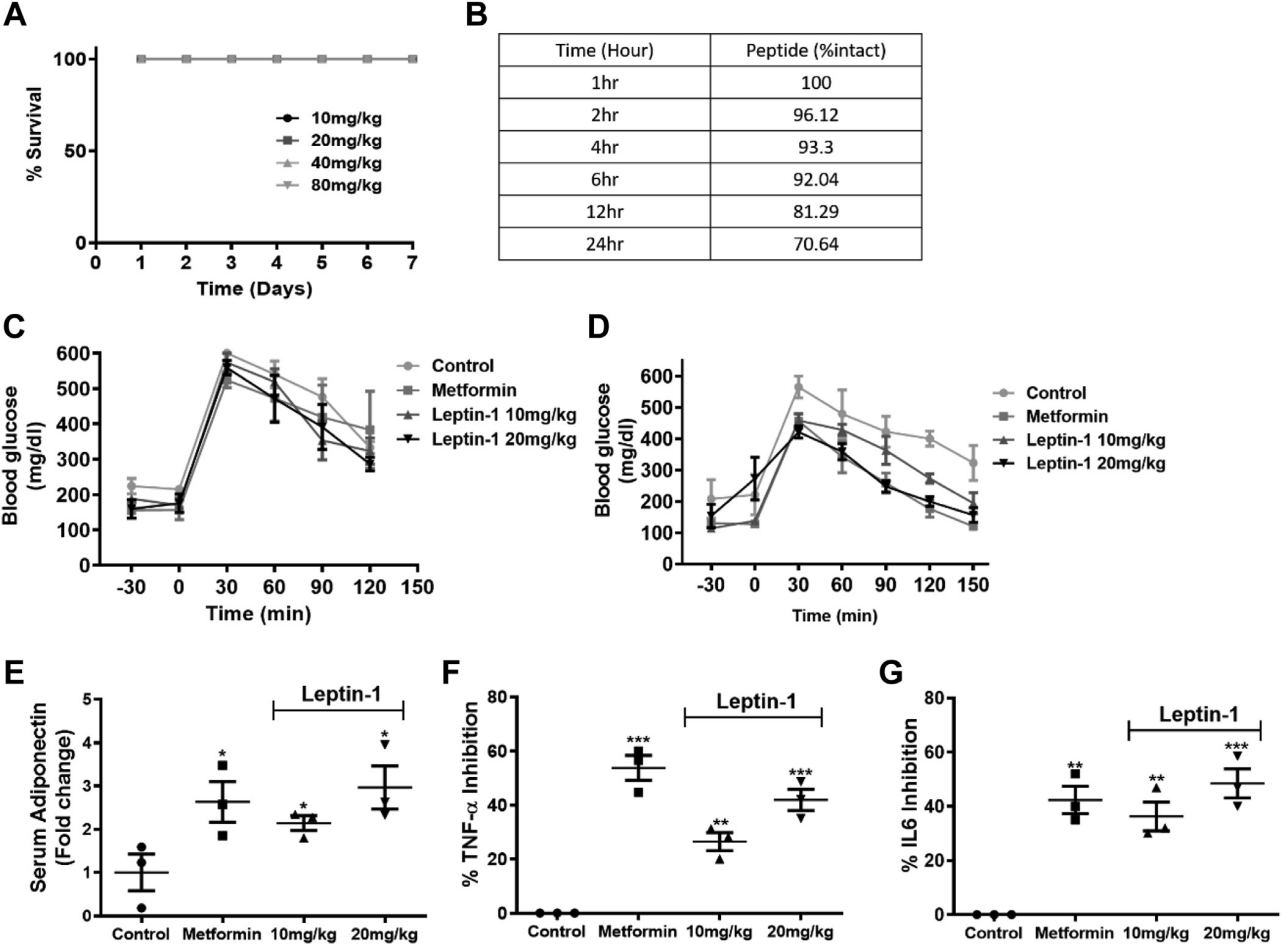
**Leptin-1 improved glucose metabolism in diabetic db/db mice.***A*, *in vivo* survival of the mice after intraperitoneally dosing with different doses of leptin-1 peptide. *B*, proteolytic stability of leptin-1 was evaluated by incubating leptin-1 in freshly prepared 10% human serum for different time periods at 37 °C. Then running the samples through HPLC column for detecting the intact peptide. *C*, OGTT histogram showing the clearance of blood glucose in db/db mice on day-1 after oral glucose loading of 3 g/kg of body weight. *D*, OGTT histogram showing blood glucose clearance in db/db mice after treatment with leptin-1 on alternative days for a period of 30 days. Leptin-1- and metformin-treated mice showed increased blood glucose clearance after 30 days of dosing. Number of mice in each group was 5 (n = 5). *E*, data showing increase in serum adiponectin level in db/db mice treated with metformin and leptin-1 compared to the vehicle treated diabetic group. *F* and *G*, serum TNFα and IL-6 levels in mice groups treated with metformin and Leptin-1. Number of mice in each group was 5 (n = 5). IL-6, interleukin 6; OGTT, oral glucose tolerance test; TNF, tumor necrosis factor.

*In vivo* toxicity of leptin-1 was examined in BALB/c mice by studying their survival and liver function at peptide doses of 10, 20, 40, and 80 mg/kg body weight for 7 days. Animals did not show any sign of toxicity and all the mice survived throughout the duration of the study, showing 100% survival (Fig. 7*A*). Serum analysis of all treatment groups showed that the serum alanine transaminase and aspartate transaminase levels were normal in all mice groups, treated with various doses of leptin-1 and were comparable to the levels in vehicle-treated control mice group (Fig. S2, *A* and *B*), suggesting no acute organ toxicity of leptin-1. Moreover, all the peptide treated mice groups maintained stable body weights until the end of 7 days experiment (Fig. S2*C*) which further confirmed the nontoxic nature of leptin-1.

### Leptin-1 peptide is serum protease stable in 10% human serum

Protease stability increases the bioavailability of a peptide in different biological systems which is conducive for its *in vivo* activity in animal. Leptin-1 showed considerable activity to induce glucose uptake in L6 myotubes which has been described in the previous sections. Further, leptin-1 did not show any significant cytotoxicity against tested mammalian cells and in mice (Figs. 1*E*, 7*A* and S2). Therefore, leptin-1 seemed to be a suitable molecule for investigating its *in vivo* activity in animal model. Thus, before investigating *in vivo* antidiabetic property of leptin-1 in mice, its proteolytic stability was examined. Since leptin-2 was significantly inactive in inducing glucose uptake in L6 myotubes as described in the previous section, its serum stability was not examined. To determine the serum protease stability of leptin-1 peptide, protease stability assays were performed in 10% human serum. Interestingly, leptin-1 showed very significant protease stability as evidenced by the recovery of more than 90% of intact peptide after incubation with human serum for 6 h by HPLC method (Fig. 7*B*). The identity of the recovered peptides was confirmed by MALDI-TOF analysis. From this observation, leptin-1 could be expected to show appreciable bioavailability in different biological systems.

### Leptin-1 peptide improved glucose metabolism in db/db mice

After assessing the *in vitro* antidiabetic activity of leptin-1 in L6 myotubes, we investigated the *in vivo* antidiabetic activity of the peptide in db/db mice. db/db mice were grouped in four different treatment groups, including a vehicle-treated negative control group, metformin-treated positive control group, leptin-1 (10 mg/kg and 20 mg/kg) treated test groups. All the animals used in this study were hyperglycemic and were intolerant to glucose exposure (Fig. 7*C*). Glucose intolerance in the animals was assessed by oral glucose tolerance test (OGTT). OGTT was performed on day one according to the protocol described in the Experimental procedure section. After performing OGTT on day-1, leptin-1 and metformin dosing were started to different treatment groups. Leptin-1 dosing was done at 10 mg/kg and 20 mg/kg of body weight, and metformin dosing was done at 50 mg/kg of body weight. Peptide and metformin at different doses were injected in intraperitoneal route in mice on alternate days. On the final day of experiment after administering peptide doses for 30 days, OGTT was again performed to determine the effect of peptide on glucose metabolism. After dosing with leptin-1 peptide for 30 days, hyperglycemia was improved and the animals showed normoglycemia in both leptin-1 treated groups at 10 mg/kg and 20 mg/kg (Fig. 7*D*) and showed improved glucose tolerance after oral glucose loading of 3 g/kg body weight. Final day OGTT data suggested that leptin-1-treated mice significantly cleared blood glucose at 120 min after 3 g/kg of oral glucose loading (Fig. 7*D*). Leptin-1 peptide dose at 20 mg/kg of bodyweight showed further improvements on both fasting blood glucose (FBG) level and glucose tolerance compared to the 10 mg/kg of body weight dosing (Fig. 7*D*). Alternate day dosing with metformin at a dose of 50 mg/kg for 30 days also reduced FBG, and improved glucose tolerance by increasing blood glucose clearance after 3 g/kg of oral glucose loading (Fig. 7*D*). Thus, these data suggested that leptin-1 significantly lowered FBG level and improved glucose tolerance in diabetic, db/db mice in a dose-dependent manner after alternate days dosing for 30 days.

### Leptin-1 increased serum adiponectin level in db/db mice suggesting improved insulin sensitivity

Adiponectin is an important adipokine hormone which improves insulin sensitivity and glucose tolerance. Thus, we determined whether leptin-1 increases the production of adiponectin in db/db mice after dosing with peptide for 30 days. For this study, blood samples were collected from the mice of different treatment groups and serum samples were isolated for biochemical analysis. Serum adiponectin levels were then estimated by ELISA assays in serum samples collected from the mice of all treatment groups. Data suggested that adiponectin production increased dose dependently in db/db mice with treatment of leptin-1 peptide at 10 mg/kg and 20 mg/kg of body weight (Fig. 7*E*). The increase in adiponectin production was presented in terms of the number of fold increase as compared to the negative control, diabetic db/db mice group. Leptin-1 dose at 20 mg/kg body weight showed more increase (3-folds) in adiponectin production as compared to the 10 mg/kg (2-folds) treated db/db mice group (Fig. 7*E*). Metformin 50 mg/kg increased adiponectin production by 2.6-folds (Fig. 7*E*). Thus, these data suggested that leptin-1 significantly increased the production of adiponectin in db/db mice and thereby increased glucose metabolism.

### Leptin-1 peptide inhibited the production of inflammatory cytokines in db/db mice

Proinflammatory cytokine levels increases in diabetic condition which impair glucose metabolism by causing IR. Inflammation and IR are linked to each other (34, 35). Therefore, we investigated the effect of leptin-1 peptide on the levels of cytokines, tumor necrosis factor alpha (TNFα) and interleukin 6 (IL-6) in db/db mice. The levels of TNFα and IL-6 in db/db mice were estimated by ELISA assays in their blood serums. Data suggested that leptin-1 treatment at 10 mg/kg and 20 mg/kg to db/db mice for 30 days inhibited TNFα production by 31% and 48%, respectively (Fig. 7*F*), whereas IL-6 production was also significantly inhibited by 46% and 56%, respectively (Fig. 7*G*). While, metformin treated db/db mice group showed the inhibitions of TNFα and IL-6 by 44% and 52%, respectively (Fig. 7, *F* and *G*). Therefore, from these observations, it can be inferred that leptin-1, derived from the N-terminal of leptin both directly and indirectly improved insulin-sensitivity and reduced IR and thus showed positive impacts on glucose metabolism in diabetic, db/db mice. However, the other peptide, leptin-2, derived from the C-terminal of leptin was much less active than leptin-1.

## Discussion

Considering the role of different regions of human LH and its critical amino acid residues in its receptor activation and biological activities two short peptides were identified, synthesized (Fig. 1, *A*–*C*), and characterized. We identified, a 17-mer (a.a, 33–49) peptide from the N-terminal loop, between helix A and helix B and a 16-mer peptide (a.a.123–138) peptide from the C-terminal helix D of LH, respectively. Our results indicate that leptin-1 and leptin-2 peptides are nonhemolytic to hRBCs and nontoxic to L6 cells (Fig. 1, *D* and *E*); leptin-1 increased glucose uptake in L6 myotubes which was associated with GLUT4 translocation to the PM of these cells (Figs. 2 and 3) and was mediated through the activation of PI3K/AKT and AMPK signaling axis (Figs. 4*D* and 5*A*). Leptin-1 exerted its metabolic effects through the activation of JAK2 protein implicating the activation of LR (Fig. 4*A*); it inhibited ACC2 by phosphorylating its serine-80 residue (Fig. 5*D*); leptin-1 increased mitochondrial biogenesis in L6 myotubes (Fig. 6, *A* and *B*) implicating its role in energy homeostasis which was further supported by leptin-1-induced expression of PGC1α, NRF1, and Tfam proteins (Fig. 6, *C*–*F*) that are important regulators of mitochondrial biogenesis and was confirmed by leptin-1-induced ATP production in L6 myotubes (Fig. 6*G*). *In vivo* animal studies demonstrated that leptin-1 improved glucose metabolism in db/db mice (Fig. 7, *C* and *D*) and it increased serum adiponectin level (Fig. 7*E*) in db/db mice suggesting that leptin-1 improves insulin sensitivity. On the other hand, leptin-2 derived from the C-terminal helix D of LH was either inactive or weakly active in all these assays.

For any antidiabetic molecule, efficient glucose lowering effect in blood or uptake of glucose in the cells is a crucial desired activity. Glucose uptake into the cells is terminally facilitated by GLUT4 protein. Corroborating with increase in glucose uptake (Fig. 2), we observed by both Western blotting (Fig. 3, *A*–*C*) and ICC (Fig. 3*D*) assays that translocation of GLUT4 to PM of L6 myotubes increased significantly in presence of leptin-1. Besides, leptin-1 also enhanced GLUT4 expression in L6 myotubes (Fig. 3, *E* and *F*). Leptin-2, derived from the C-terminal of LH did not show any significant effect on GLUT4 expression in L6 myotubes (Fig. 3, *E* and *F*). This difference between the ability of the N-terminal derived leptin-1 and C-terminal derived leptin-2 in increasing glucose uptake and GLUT4 expression in L6 myotubes supports the previous findings that the N-terminal of LH is more important than its C-terminal region for binding to LR and its activation (30). The results also justify our hypothesis on identifying a peptide from the N-terminal of leptin with metabolic property.

While investigating the mechanism of action, we observed that leptin-1 initiated its signaling pathway through LR which was established by the evidence that leptin-1 activated JAK2 protein (Fig. 4, *A* and *B*), which is the immediate downstream target protein of LR. As mentioned in the results section of “identification of leptin-1 and leptin-2 peptides” that leptin-1 and leptin-2 possess multiple amino acid residues that could participate in the interaction of the concerned peptide with its target protein. Yet, leptin-1 showed stronger JAK2 activation than leptin-2 implicating its possible interaction with LR. The results could be implied that interaction of leptin-1 with LR is specific for the primary structure of this peptide which corroborates with the findings of earlier mutagenesis study (30).

We observed that leptin-1 increased the expressions of IRS1, PI3K, and AKT proteins (Fig. 4, *A*, *C–F*) in L6 myotubes. Additionally, we observed that activation of p-AKT by leptin-1 was inhibited in the presence of PI3K inhibitor wortmannin (Fig. 4, *G* and *H*) which confirmed that activation of p-AKT by leptin-1 is mediated through activation of PI3K. That leptin-peptide, leptin-1 activates IRS1 which plays crucial roles in glucose metabolism, signifies that this peptide could exert positive effects on diabetes.

Results indicated that leptin-1 activated PI3K/AKT and AMPK proteins (Figs. 4*D* and 5*A*), which are key regulators of glucose metabolism. Furthermore, leptin-1-induced glucose uptake in L6-myotubes was inhibited in presence of inhibitors of PI3K, AKT, and AMPK proteins, suggesting that leptin-1-induced glucose uptake was mediated through these proteins (Fig. 5*C*). In addition to the involvement in glucose metabolism, AMPK also contributes in energy homeostasis. The critical pathways activated by AMPK that are involved in energy homeostasis are, inhibition of ACC2, activation of mitochondrial biogenesis, and ATP production. In accordance to these, we observed that leptin-1 induced phosphorylation of ACC2 at S80 residue (Fig. 5, *D* and *E*) which inhibits the activity of this protein (20). Additionally, we also observed that phosphorylation of ACC2 at S80 residue by leptin-1 was inhibited in the presence of dorsomorphin which indicates that ACC2 phosphorylation in the presence of the peptide is mediated through AMPK activation (Fig. 5, *F* and *G*). As mentioned earlier, AMPK is considered as the energy sensor of a cell. Along with the activation of AMPK, leptin-1 also increased mitochondrial biogenesis in L6 myotubes (Fig. 6, *A* and *B*). Further signifying the biogenesis of mitochondria, we also observed that leptin-1 increased the expression of PGC1α, NRF1, and Tfam proteins in L6 myotubes (Fig. 6, *C*–*F*). These proteins are important regulators of mitochondrial biogenesis and regulate expressions and activations of genes involved in the mitochondrial DNA replication and its biogenesis (24, 25). However, leptin-2 was much less active than leptin-1 in inducing mitochondrial biogenesis (Fig. 6, *A* and *B*) and activation of PGC1α, NRF1, and Tfam (Fig. 6, *C–F*) in L6 myotubes.

After investigating the *in vitro* antidiabetic activity of the leptin-1 and leptin-2 peptides in L6 myotubes, *in vivo* antidiabetic activity of the significantly active peptide, leptin-1 in diabetic animal model db/db mice was also examined. The experiment was planned considering the significant nontoxic nature of leptin-1 in both *in vitro* (Fig. 1, *D* and *E*) and *in vivo* studies (Fig. 7*A*) as well as its appreciable stability in the presence of human serum (Fig. 7*B*). Dosing with leptin-1 peptide at 10 mg/kg and 20 mg/kg body weight to diabetic, db/db mice for 30 days on alternate day basis improved hyperglycemia and significantly lowered the fasting blood glucose level (Fig. 7*D*). Improvement in the measured parameters was confirmed by comparing these values with the vehicle treated diabetic control group (Fig. 7*C*). Diabetic, db/db mice are highly intolerant to glucose exposure and showed abnormal blood glucose clearance when an oral glucose dose (3 g/kg body weight) was given to these animals. Proinflammatory cytokines disturb glucose metabolism and inversely affect the adiponectin level and insulin sensitivity. Remarkably, leptin-1 treatment increased adiponectin secretion (Fig. 7*E*) and inhibited TNF-α and IL-6 production (Fig. 7, *F* and *G*) in db/db mice resulting in the improvement of insulin sensitivity and attenuation of IR.

Overall, to our knowledge, the current study is the first report of its kind that shows the identification and characterization of a human leptin-derived peptide which improves glucose metabolism and energy homeostasis by enhancing glucose uptake, mitochondrial biogenesis, and the production of ATP. Human leptin N-terminal AB loop derived, 17-mer leptin-1 peptide significantly improved glucose metabolism in both *in vitro* and *in vivo* studies. Leptin-1 also showed positive effects on mitochondrial biogenesis and ATP production. Further, it increased adiponectin secretion and inhibited proinflammatory cytokine productions in diabetic, db/db mice. The current investigation supports the earlier mutagenesis studies which indicated the crucial role for N-terminal region of the protein in its receptor binding and activation and thus in its biological activities. In contrast to the versatile metabolic properties of leptin-1, a similar sized (16-mer) human leptin C-terminal helix D-derived, leptin-2 peptide was much less active. Thus taking clues from an earlier study on mutagenesis of leptin and other related studies in the literature, we have identified a human leptin-derived peptide that possesses significant properties of the protein. Overall, the current study could aid in the investigations for identifying new therapeutic peptides with metabolic properties of leptin.

## Experimental procedures

### Materials

#### Cell lines and animals

Cell lines purchased from American Type Culture Collection, L6 cell line (CRL-1458). db/db and BALB/c mice used in this study were provided by Laboratory Animal Facility, CSIR-CDRI.

#### Antibodies and assay kits

Antibodies used were purchased from Cell Signaling Technology unless otherwise mentioned. Mouse anti-GLUT4 (IF8) (sc53566) and anti-Tfam (F-6) (sc-166965) antibodies were purchased from SantaCruz, Rabbit anti-GLUT4 purchased from Abcam (ab654), Anti-IRS1 (05-1085), Anti-PGC1α (ST1203),and Anti-AKT (05-591) purchased from millipore. Anti-pAKT pS473 (9271S), Anti-PI3K p85 (4292S), Anti-pAMPK (2535S), Anti-AMPK (2532S), Anti-NRF1 (46743S), Anti-pACC (3661S), Anti-ACC(3676T), Anti-pJAK2 (3776S), Anti-β-Actin (8457S), and Alexa flour-488 labeled Rabbit secondary antibody was purchased from Cell Signaling Technology. Glucose uptake kit (ab136955) purchased from Abcam. Mouse Adiponectin (ITEM0001) ELISA kits were purchased from ImmunoTag. Mouse TNF alpha (558534) and IL6 (555240) ELISA assay kits were purchased from BD Biosciences.

#### Chemicals

Cell culture media, Dulbecco's modified Eagle's medium (DMEM)-high glucose (D7777) and low glucose (D5523) purchased from Sigma-Aldrich. RPMI-1640 (11875-093), fetal bovine serum United States origin (16000-044), Antibiotic-Antimycotics 100× (15240-062) were purchased from Thermo Fisher Scientific. 2-(7-Nitrobenz-2- oxa-1, 3-diazol-4-yl) amino-2-deoxy-D-glucose (2NBDG) (11046), wortmannin (10010591), AKTi (14870), indinavir and dorsomorphin (11967) from Cayman. Protease inhibitor cocktail (P8340), phosphatase inhibitor cocktail 2 and 3 (P5726 and P0044), human insulin (91077C), rosiglitazone (R2408) and 3-(4,5-dimethylthiazol-2-yl)-2,5-diphenyltetrazolium bromide (MTT) were purchased from Sigma-Aldrich, Metformin from MP Biomedicals. Mitotracker red (M46752) and Hoechst (H21486) were purchased from Invitrogen. Rink amide resin 200mesh, fluorenylmethoxycarbonyl (Fmoc)-protected amino acids, HCTU and oxyma pure were purchased from Novabiochem. 5,6-carboxytetramethylrhodamine N-succinimidyl ester (5,6-TAMRA, SE), 4-methylmorpholine, phenol, and potassium cyanide were purchased from Sigma-Aldrich and ninhydrin from SRL. Peptide synthesis solvents dimethylformamide, dichloromethane, piperidine, and diethyl ether were purchased from Spectrochem. HPLC grade solvents, acetonitrile, and methanol were purchased from Merck Millipore.

## Methods

### Synthesis and purification of peptides

Peptides were synthesized by solid phase peptide synthesis method by using the Fmoc chemistry, following the procedure described earlier (36). PS3 peptide synthesizer from Protein Technologies was used to synthesize the peptides. The synthesized peptides were cleaved from the resin using a cleavage mixture of Thioanisole: P-cresol: ethanedithiol in ratio of 1:1:0.5 under acidic condition in trifluoroacetic acid. The crude peptide was than purified by Waters reverse phase-HPLC using a C-18 column. A 90%: 10%, water: acetonitrile gradient program of 40 min with 2 ml/min flow rate was run to elute the peptides. The confirmation of the synthesized peptide was done by determining the mass/charge ratio by MALDI-MS analysis (37). The peptides were than lyophilized to powder and stored at −20 °C.

### Cell culture

#### L6 rat myoblast cell line

Biological activity and toxicity of the purified peptides were studied on rat skeletal muscle cell line L6. L6 cell line was seeded, grown, and differentiated according to the protocols described earlier (34). In brief, cells were grown in low glucose-DMEM and differentiated using DMEM containing 2% fetal bovine serum supplemented 1% Antibiotic-Antimycotic (38, 39).

### Animals

db/db mice procured for this study were 5 to 6 weeks old and weighs 45 ± 5 g in body weight. BALB/c mice were 5 to 6 weeks old and weighs 20-25 g body weight. All the studies were performed on male mice. Animals were kept in CDRI animal house facilities under standard temperature, relative humidity, and 12 h day/night condition. On arrival, animals were allowed to acclimatize for a week before starting the experiments. Chow diet and drinking water was all time accessible to the animals and fasting of the animals was done whenever required in the study. This animal study was carried out with CDRI animal ethics committee approval (IAEC/2022/124) and all the guidelines framed by Committee for the Purpose of Control and Supervision of Experiments on Animals were properly followed.

### Hemolytic and cell viability assay of the peptides

Toxicity of the peptides was studied against hRBC and L6 cell line, according to the protocols mentioned earlier (34). For hemolytic assay, blood sample was collected from healthy human volunteers with the approval of the institutional ethics committee, bearing approval No. CDRI/IEC/2019/A1 dated 11th June 2019. Further, the studies abide by the Declaration of Helsinki principles, and all the subjects were given written informed consent. Briefly describing, for hemolytic assay, blood sample was collected from a healthy volunteer and RBCs were isolated. Subsequently, 4% suspension of RBCs were incubated with peptides at different doses for 45 min with gentle shaking. Absorbance of the supernatant was measured at 540 nm for presence of hemoglobin released due to hemolysis. To study cytotoxicity, L6 cells seeded in 96-well plate were incubated with peptides at different doses for 2 h. The cells were then treated with MTT for 2 h. MTT solution was then aspirated, and the MTT into the cells were extracted using 100% dimethyl sulfoxide and the absorbance was measured at 540 nm.

### Glucose uptake assay

As described earlier (34), glucose uptake assay was performed in L6 myotubes using 2NBDG. Briefly describing, L6 cells were seeded and differentiated to myotubes as described above. Differentiated myotubes were serum fasted for 6 h with serum free DMEM and the cells were incubated with desired concentration of leptin-1 and leptin-2 peptides for specified period. Positive control insulin treatment was given at a concentration of 100 nM for 30 min. Following peptide treatment, the cells were incubated with 200 μM of 2NBDG for 10 min and the residual 2NBDG was removed and washed with PBS. Cells were trypsinized and collected in fluorescence activated cell sorting tube for fluorescence measurement. To estimate the uptake of fluorescent glucose analog, fluorescence of the cells from different samples was measured using fluorescence activated cell sorting Aria instrument.

### Cytosolic and membrane fraction isolation

Separation of cytosolic and membrane fraction was performed according to the protocol mentioned earlier (34). In brief, the harvested cells were resuspended in membrane fractionation buffer (40) and kept for 1 h with regular tapping. Cellular debris were removed by centrifugation at 800*g* for 10 min, and the supernatant was collected in fresh micro centrifuge tube, which contains the cytosolic and crude membrane fractions. Crude membrane and cytosolic fraction were separated by centrifugation at 12000 rpm for 10 min at 4 °C. The crude membrane pellet was resuspended in radioimmunoprecipitation assay lysis buffer to elute out membrane proteins and then centrifuged at 15000 rpm for 15 min. Whole process was performed on ice. Both the samples were than resolved on 10% SDS-PAGE (38, 40).

### Whole cell lysate preparation, SDS-PAGE, and Western blotting

In order to examine the peptide-induced activation of a protein, related to a signaling event, we have assessed its expression in the peptide-treated L6 myotubes with respect to the myotubes, not treated with the peptide and myotubes-treated with a positive control. For this purpose, we have performed Western blotting experiments by usual procedure after resolving the proteins of the whole cell lysates of L6 myotubes, treated at different experimental conditions by SDS-PAGE. Briefly describing the procedure, whole cell lysate was prepared by lysing the cells in radioimmunoprecipitation assay lysis buffer and equal amount of lysate was resolved on 10% SDS-PAGE. Resolved proteins were transferred to nitrocellulose membrane and the membrane was blocked with 5% skimmed milk solution. Incubated with specific primary (ratio of 1: 2500 of antibody to phosphate-buffered saline with Tween 20 (PBST) used for antibodies purchased from Sigma-Aldrich and Millipore, 1:1000 for antibodies purchased from Santa Cruz) and secondary antibodies (antibody concentration of 1:2000 in PBST), and the blots were developed using ChemiDoc (34).

On the other hand, for examining the peptide-induced translocation of GLUT4 into the PM of L6 myotubes, we have collected the cytosolic and crude PM fractions from peptide-treated L6 myotube extract as described in the previous section. Then after resolving the proteins in each fraction by SDS-PAGE, GLUT4 was probed by Western blotting.

### Immunocytochemistry

ICC analysis for GLUT4 translocation to PM was studied in L6 myotubes. L6 cells were grown and differentiated in 4-chambered slide following the protocol described above. The differentiated cells were treated with test peptide and positive control insulin for specified time period. Cells were fixed in formalin and perforated using 0.4% Triton X-100. The cells were then immunostained using anti-GLUT4 primary antibody (1:100 dilution) and incubated overnight at 4 °C. Cells were washed three times with PBST and then incubated with Alexa flour-488 tagged secondary antibody and incubated for 2 h. After 2 h, cells were again washed 5 times with PBST, and nuclear staining was performed by incubating the cells for 30 min with Hoechst nuclear stain. The cells were then again washed 3 times and the slide was mounted using DPX mounting media. Imaging of the slides was done using confocal microscope, blue range and green ranges filters were used (34).

### Mitochondrial biogenesis assay using Mito Tracker red

Mitochondrial biogenesis was studied in L6 cells using mitochondrial stain MitoTracker CMXRos. The cells L6 was seeded in 4-chambered slide with a density of 20,000 cells per chamber. The cells were differentiated to myotubes following the protocol mentioned above. On differentiation, the cells were treated with desired concentration of test peptide and positive control rosiglitazone (10 μM) for specified period of 48 h. Following treatment with test peptides the cells were stained with MitoTracker red and Hoechst. The cells were then washed with PBS to remove the residual stains. Cells were then fixed with 4% paraformaldehyde and the slides were mounted using DPX. The slides were then imaged using confocal microscope. The excitation and emission wavelengths were fixed at 578 and 599 nm for red and 357 and 538 nm for blue emission, respectively (41).

### ATP extraction and determination

Whole amount of ATP was extracted from L6-myotubes treated with leptin-1 and leptin-2 peptides and Rosiglitazone, according to the protocol mentioned in the literature (42). Briefly, the cells were harvested and pelleted by centrifugation. The harvested cells were incubated with 1% trichloroacetic acid and 4 mM EDTA solution for 10 min on ice for ATP extraction. The samples were centrifuged at 12,000 rpm for 10 min to pellet down the debris, and the supernatant was used for ATP determination. ATP determination was performed according to the protocol mentioned in the ATP determination kit’s manual from Thermo Fisher Scientific. Interference of trichloroacetic acid on ATP determination was avoided by 10× dilution of the sample in water.

### Serum protease stability

Protease stability of the leptin-1 peptide was determined in 10% human serum. This study was performed with the approval from the institutional ethics committee, bearing the approval number Blood sample from healthy volunteer was collected and allowed to clot. The clotted blood sample was then centrifuged to pellet down the clot, and the supernatant containing the serum was collected in fresh micro centrifuge tubes. From the serum isolated, 20% working serum solution in Milli-Q water was prepared. Briefly, 100 μg of peptide was incubated in an equal volume of 20% human serum at 37 °C for 0, 15, 30, 45, 60, and 120 min. After incubation, chilled methanol was added to the reaction mixture and pelleted down the serum protein precipitates. Subsequently, 100 μl of the supernatant was then injected to the C-18 HPLC column to detect the peptide peak in HPLC chromatogram. A separate HPLC chromatogram for only peptide and serum was also obtained to differentiate the HPLC peak of serum protein and the leptin-1 peptide in the chromatogram. The only peptide HPLC peak was considered as the peak corresponding to the 100% intact peptide. The HPLC peak in the only serum containing chromatogram was not included in the analysis. Percentage recovery of the peptide incubated with 10% human serum for different time interval was calculated by comparing with the only peptide containing HPLC chromatogram (38).

## Animal studies

### *In vivo* acute toxicity and survival study in BALB/c mice

*In vivo* toxicity and survival study with the peptides were performed in BALB/c mice. Mice were divided into five groups, each group with five number of mice. One group was kept as control group and injected only with vehicle (Water). Four other groups were injected with different doses of M10 peptide at 20, 40, 80, and 100 mg/kg body weight. Dosing was done through intraperitoneal route and only a single dosing was performed. The mice were kept under observation for a week after peptide injection. After 1 week of observation, survival of mice in all groups was recorded. At seventh day, blood samples were collected from each mice in all the groups, and the serum was isolated to estimate aspartate transaminase and alanine transaminase level (43).

### *In vivo* antidiabetic activity study in db/db mice

*In vivo* antidiabetic activity of the peptide was studied in diabetic animal model db/db mice. Male db/db mice with hyperglycemia were identified by checking their blood glucose level and selected for the study. Mice were divided into four groups, and the number of animals in each group was five. Blood glucose levels were measured using Accu-Chek glucometer and blood glucose measurement strips. The study was continued for 30 days. At the start of the study, fasting blood glucose in each animal was recorded, and OGTT was performed in each animal of all groups. To perform OGTT, the mice were fasted for 6 h, and then 30 min before oral glucose loading, the fasting blood glucose level in each mouse was recorded. After fasting of 6 h 3 g/kg body weight glucose was loaded orally and blood glucose at 0 minute was recorded simultaneously. Blood glucose was measured after every 30 min at 30, 60, 90, and 120 min after oral glucose loading. Time of oral glucose loading was considered as 0 min. After OGTT on day-1 peptide dosing was started. Dosing of M10 peptide at 10 mg/kg and 20 mg/kg body weight was started from day one on alternate day basis through intraperitoneal route for 30 days. Thus 15 doses of the peptide/drug were administered in each db/db mice over a period of 30 days. On final day, OGTT was performed again with the same procedure described above. Blood sample was collected from each group for serum analysis. Mice were sacrificed by CO2 induced hypoxia. Tissue samples were also collected from all group of mice and fixed in formalin for histopathological examination.

### Biochemical analysis of serum

Blood sample from all group of animals were collected through retro orbital sinus and were allowed to clot at room temperature for 1 h. The sample was centrifuged at 3000 rpm for 20 min at 4 °C, and serum was collected in fresh microcentrifuge tubes. Collected serum was either freshly used for biochemical analysis or stored at −20 °C for future use. Biochemical analysis of the serum samples for metabolic hormones and cytokines was performed by ELISA, following the manufacturer’s protocol given in the manual.

### Statistical analysis

Each of the experiments has been repeated at least three times. Statistical analysis of data was done using GraphPad prism software (https://www.graphpad.com/features#analysis). All phosphoproteins were quantified by normalizing with their total proteins. All data are presented as means ± SD. *p* ≤ 0.05 was considered significant.

## Data availability

All the data required for this manuscript are available either in the main article or in the supplementary information file.

## Supporting information

This article contains supporting information.

## Conflict of interest

The authors declare that they have no conflicts of interest with the contents of this article.
