# Supplementary material for: A peptide derived from the amino terminus of leptin improves glucose metabolism and energy homeostasis in myotubes and db/db mice
Source: J Biol Chem. 2024 Oct 28;300(12):107919. doi: 10.1016/j.jbc.2024.107919 (PMC11625344; doi:10.1016/j.jbc.2024.107919)
Supplement: Supplemental Figures S1 and S2 [file mmc1.docx]

**A peptide derived from the amino terminus of leptin improves glucose metabolism and energy homeostasis in myotubes and db/db mice**

**Mehmood Ali ^1,2^, Arvind Gupta ^1,2^, Rahul Dev Verma ^1,2^, Sariyah Akhtar ^1^, Jimut Kanti Ghosh ^1,2*^.**

**Running title: Metabolic effects of Leptin amino terminus-derived peptide**

**Materials Included**

Figure-S1

Figure-S2


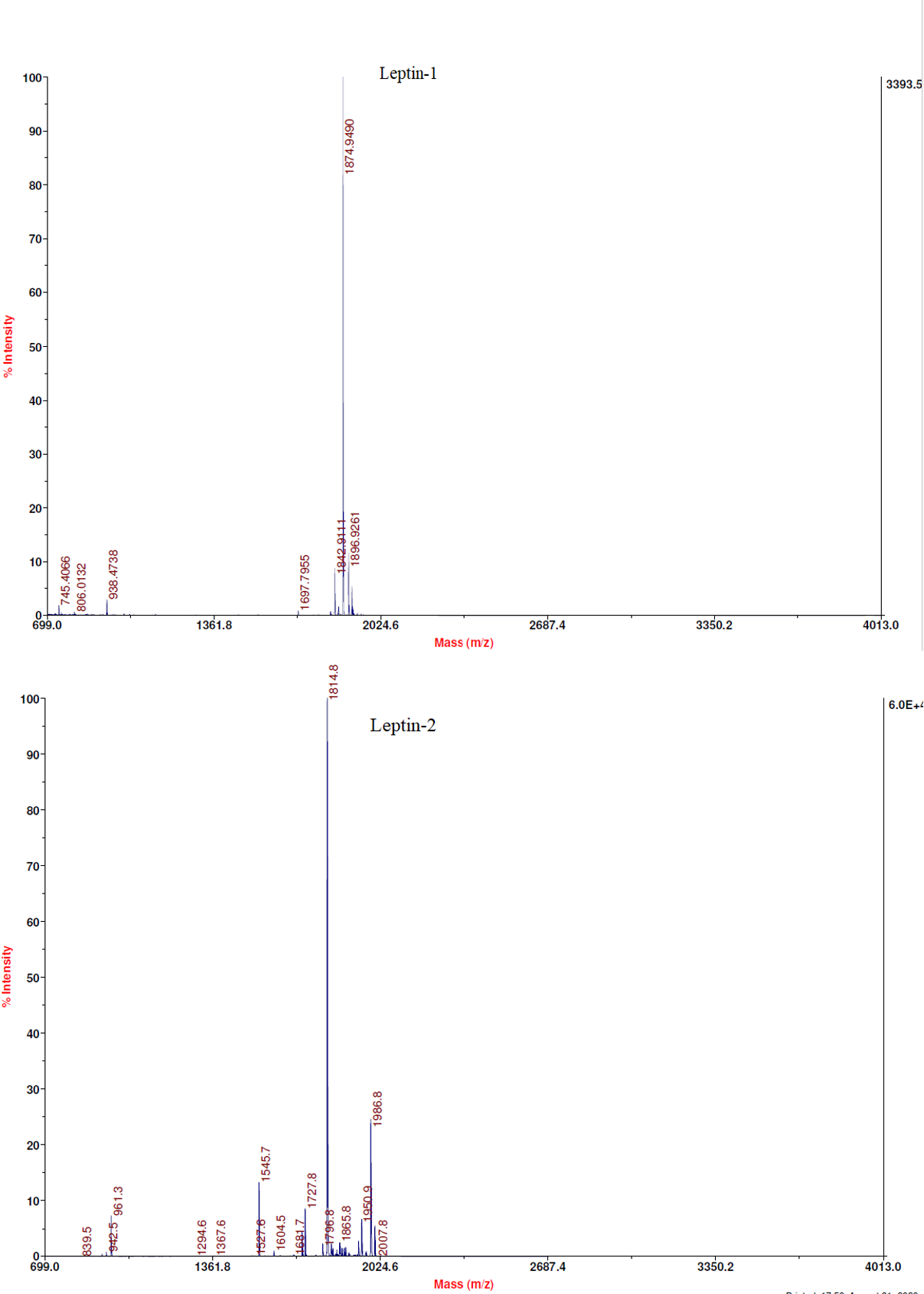


**Figure-S1**: MALDI-MS spectra of Leptin-1and Leptin-2 peptides.


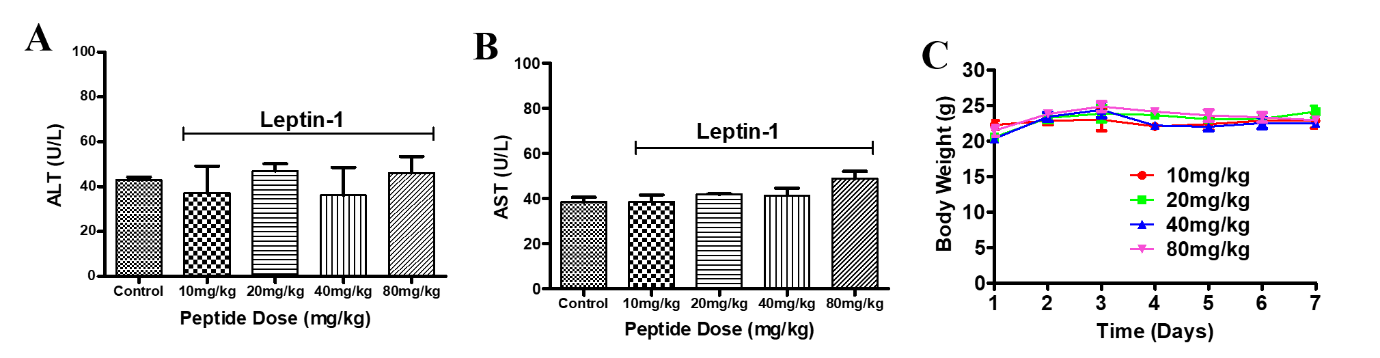


**Figure-S2: In-vivo toxicity study: A,** *In-vivo* survival of the mice after intraperitoneally dosing with different doses of Leptin-1 peptide. **B and C.** Showing serum Alanine Transaminase (ALT) and Aspartate Transaminase (AST) level in mice treated with Leptin-1 peptide. **F.** Body weight of the mice during the 7-days after treating with Leptin-1 peptide.
